# Multiple Roles of Brd4 in the Infectious Cycle of Human Papillomaviruses

**DOI:** 10.3389/fmolb.2021.725794

**Published:** 2021-07-27

**Authors:** Alison A. McBride, Alix Warburton, Simran Khurana

**Affiliations:** Laboratory of Viral Diseases, National Institute of Allergy and Infectious Diseases, National Institutes of Health, Bethesda, MD, United States

**Keywords:** bromodomain-containing protein 4, cancer, human papillomavirus, replication, transcription, DNA damage response, virus, papillomavirus

## Abstract

Human Papillomaviruses (HPV) reproduce in stratified epithelia by establishing a reservoir of low- level infection in the dividing basal cells and restricting the production of viral particles to terminally differentiated cells. These small DNA viruses hijack pivotal cellular processes and pathways to support the persistent infectious cycle. One cellular factor that is key to multiple stages of viral replication and transcription is the BET (bromodomain and extra-terminal domain) protein, Brd4 (Bromodomain containing protein 4). Here we provide an overview of the multiple interactions of Brd4 that occur throughout the HPV infectious cycle.

## Papillomavirus Diversity and Evolution

Papillomaviruses are small DNA viruses that cause persistent infection of the skin and mucosa of bony vertebrates. To date, the genomes of ∼665 individual papillomavirus types have be sequenced and characterized (https://pave.niaid.nih.gov/). The viruses are genetically stable and have coevolved with their hosts for millions of years (Van Doorslaer, 2013); each virus is species specific and often tropic for specific anatomical regions of the cutaneous or mucosal epithelia of the host. The viruses have been shaped by evolution for millions of years and the availability of DNA and protein sequences for each can help decipher the role of specific viral-host interactions in viral-associated disease. Here we review how the E2 proteins of all papillomaviruses examined to date interact with the host protein, Brd4. Yet, the specific nature of these interactions differs among different viral types; understanding these nuances will help elucidate the precise role of Brd4 in papillomavirus infection and associated disease.

## Papillomavirus Infectious Cycle

**FIGURE 1:**
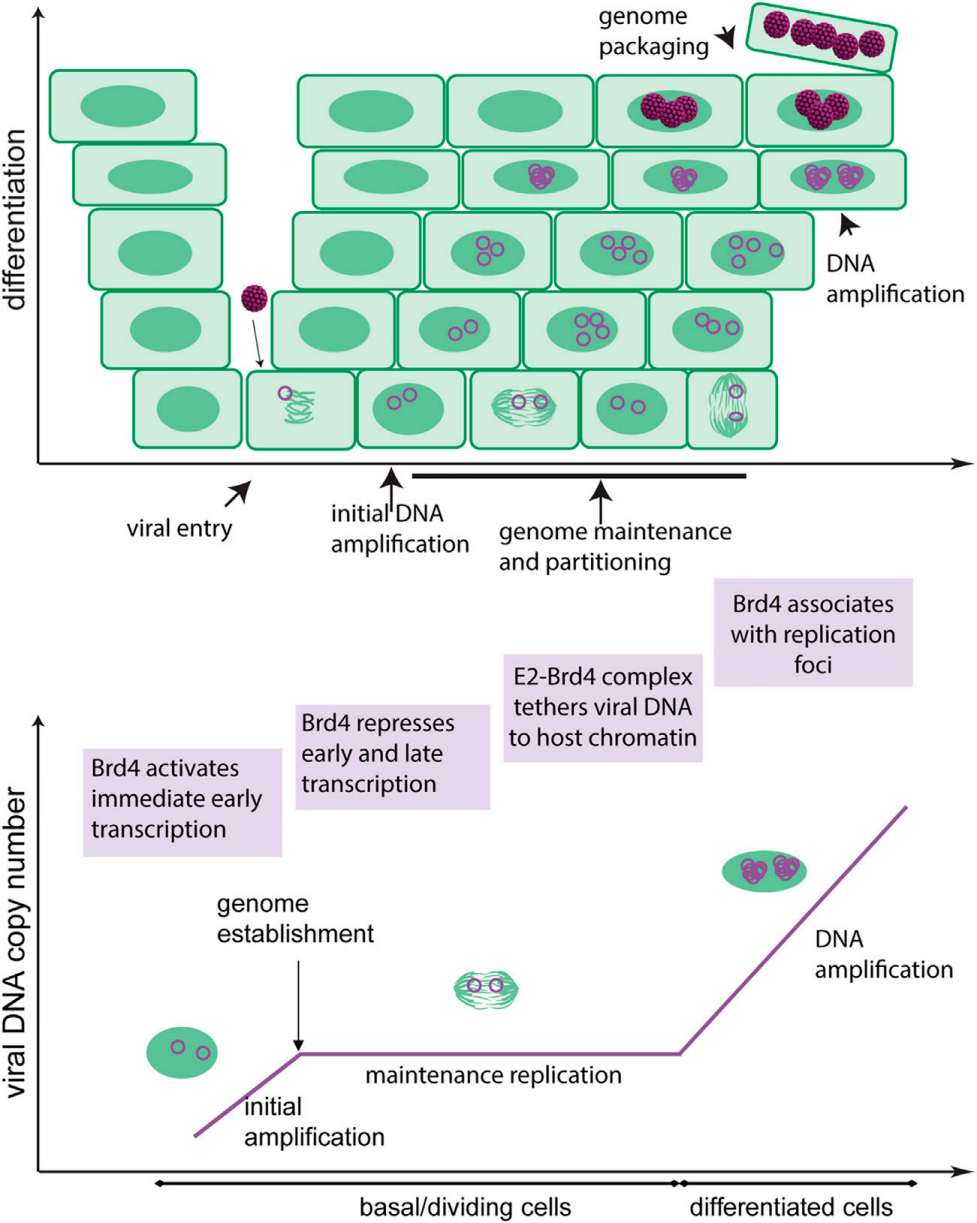
Roles of Brd4 in the HPV infectious cycle. The upper diagram shows the differentiated layers of a stratified epithelium. HPV accesses the basal cells through a microabrasion where it initiates a persistent infection. The viral genome is maintained as an extrachromosomal element within these cells and is partitioned to daughter cells by association with host chromosomes. As the infected cells differentiate, the viral genome amplifies to a high copy number in nuclear foci and is subsequently packaged into virions. The virions are shed from the surface of the epithelium in squames. The lower diagram shows the different stages of viral replication. The roles of Brd4 in the HPV infectious cycle are shown in purple boxes.

Papillomaviruses replicate persistently in the stratified mucosal and cutaneous epithelia of their host. The virus enters the dividing basal cells of the epithelium through a microabrasion and after a few rounds of DNA replication, establishes its genome as a stable extrachromosomal plasmid in the nucleus of the host cell. The genome persists in the basal cells for long periods of time with only low levels of viral gene expression, which helps it escape immune detection. The basal cells of a stratified epithelium can divide symmetrically to produce new basal cells, and asymmetrically to produce a daughter cell that proceeds through the process of differentiation to the surface of the tissue. By establishing their genomes in basal cells, papillomaviruses take advantage of this process as it allows them to form a stable reservoir of low level infection in symmetrically dividing basal cells while simultaneously generating infected cells by asymmetric division that will differentiate and activate the productive stage of viral infection. The stages of the infectious cycle are depicted in Figure 1.

## Human Papillomaviruses Infection and Associated Disease

Over 440 HPV types have been identified and sequenced. They are classified into five different genera: Alpha, Beta, Gamma, Mu, and Nu (Bernard et al., 2010). Beta and Gamma viruses cause asymptomatic infection of the cutaneous epithelium and Mu and Nu cause common warts on the hands and feet (Cubie, 2013). The clinical association of Alphapapillomaviruses is more diverse and can range from common warts in the cutaneous epithelium to anogenital lesions in mucosa (Cubie, 2013). A subset of Alphapapillomaviruses are classified as oncogenic as in a small portion of cases, chronic infection with these viruses leads to cancer. Five percent of human cancers, such as cervical, anal, and oropharyngeal carcinoma, can be attributed to infection by oncogenic HPV types (Viens et al., 2016). Betapapillomaviruses are associated with asymptomatic infection and can be considered commensals in immunocompetent individuals. Nevertheless, by interfering with cellular DNA repair pathways, they may predispose to squamous cell skin cancer (Tommasino, 2019).

## Human Papillomaviruses Genome Regulation

### Papillomavirus Genome and Gene Products

**FIGURE 2:**
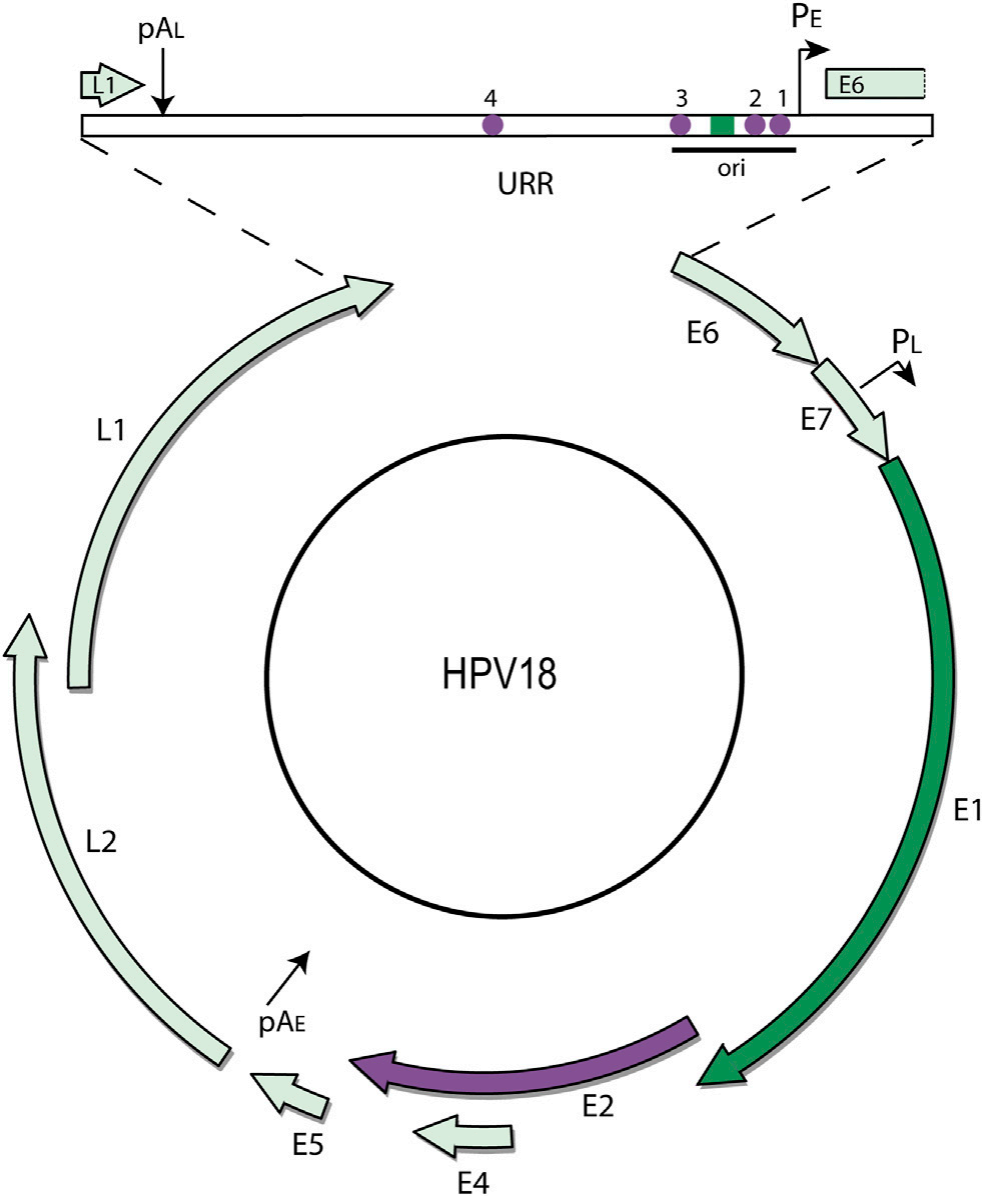
HPV18 genome map. The (8 kbp) circular dsDNA genome of HPV18 is shown. Only one strand of the DNA is transcribed, and transcription is initiated from the early and late promoters (P_E_ and P_L_) and terminated at the early and late polyadenylation sites (pA_E_ and pA_L_) as indicated. The major open reading frames are represented by arrows. The URR (upstream regulatory region) contains the replication origin and E1 and E2 binding sites. The E1 ORF and corresponding binding site is highlighted in dark green, and the E2 ORF and corresponding binding sites in purple.

All papillomaviruses have a very similar genome organization. They have circular dsDNA genomes of ∼7,000–8,000bp. The genome is organized into three regions: the Upstream Regulatory Region (URR), a non-coding region containing the replication origin and transcriptional regulatory elements; the early coding region (expressed in early stages of infection); and the late coding region (expressed only at late stages of infection). Figure 2 shows an Alphapapillomavirus genome.

There are four viral proteins that are conserved in all papillomaviruses: the L1 and L2 capsid proteins and the E1 and E2 replication proteins. The other proteins E5, E6, E7, and E4 can be considered accessory proteins that shape the cellular environment to support each stage of the viral infection (Della Fera et al., 2021). They do this by changing the balance of cellular proliferation and differentiation and by interfering with immune detection of the virus (Della Fera et al., 2021). In this review, we will focus on the E1 and E2 replication/transcription regulatory proteins that are involved in viral processes that intersect with the Brd4 protein.

### Human Papillomaviruses Transcriptional Regulation

In all papillomaviruses, only one DNA strand of the genome is transcribed. Transcription is initiated from early and late promoters located in the URR or 5’ half of the early coding region and these terminate at one of two polyadenylation sites located at the end of the early and late coding regions, respectively (Figure 2). Multiple alternatively spliced transcripts are generated from these regions and transcription is regulated extensively by RNA processing and polyadenylation site choice (Johansson and Schwartz, 2013; Schwartz, 2013).

HPV genomes, and in particular the URR, contain many binding sites for ubiquitous cellular transcription factors such as AP1, NF1, Oct1, TEF1, YY1, C/EBP, and steroid hormone receptors (Bernard, 2013). Transcription can also be repressed by a topological chromatin loop that links the URR to the early coding region in undifferentiated cells (Paris et al., 2015; Pentland et al., 2018). This loop is mediated by CTCF and YY1 and is released upon differentiation to promote viral gene expression (Pentland et al., 2018).

Papillomavirus genomes are assembled in host nucleosomes at all stages of infection, including inside virion particles (Favre et al., 1977; Porter et al., 2021). In fact, HPV chromatin is modified at all stages of the infectious cycle (Mar Pena and Laimins, 2001; Wooldridge and Laimins, 2008; Langsfeld et al., 2015; Gautam and Moody, 2016; Burley et al., 2020; Porter et al., 2021). This provides another layer of regulation mediated by histone post-translational modifications, and cellular factors that bind to modified chromatin, such as Brd4.

**FIGURE 3:**
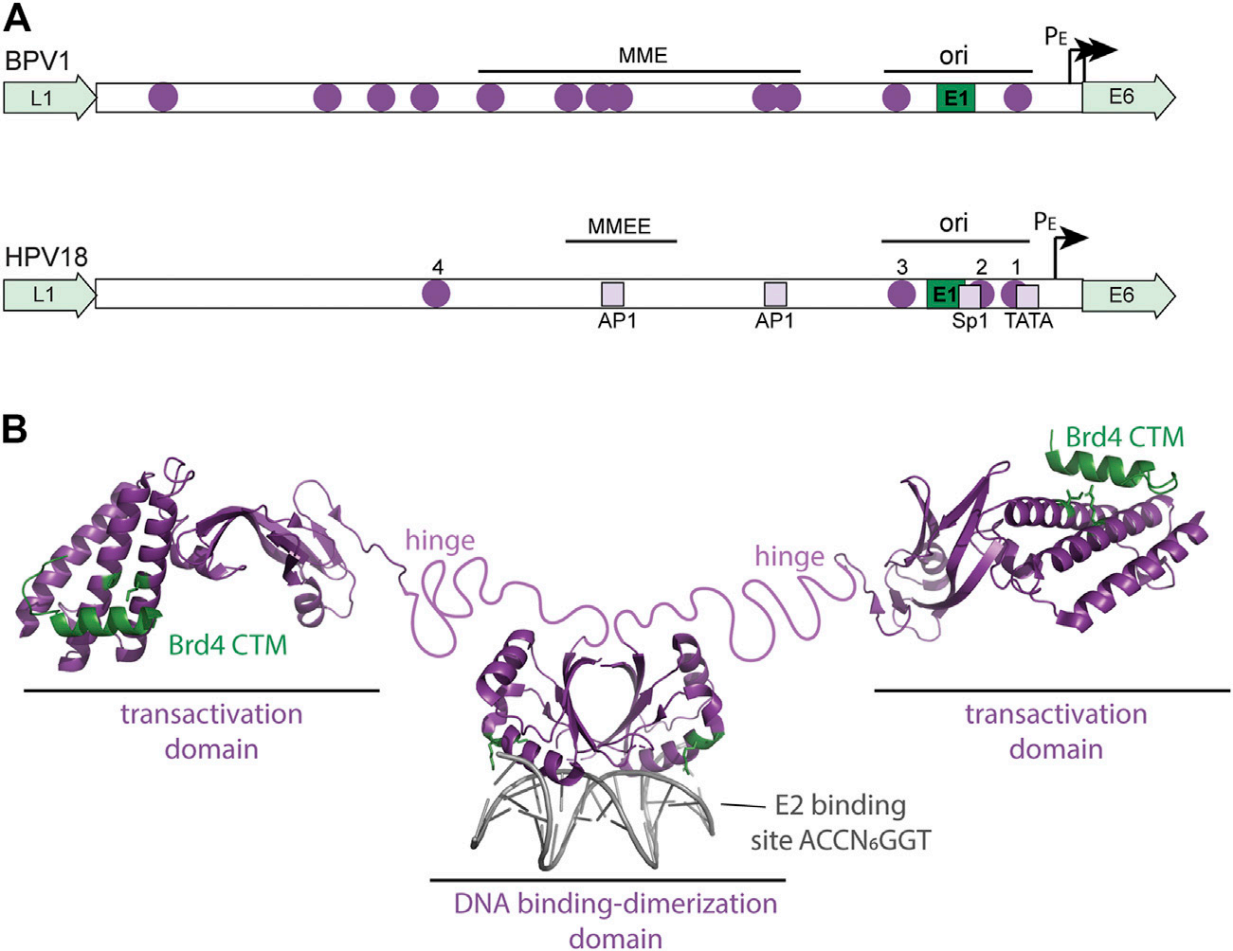
Regulatory elements in the viral URR and structure of the HPV E2 protein. **(A)** The diagram shows a comparison of the BPV1 and HPV18 Upstream Regulatory Regions (URR). Both URRs contain the replication origin (with E1 and E2 binding sites) as well as the additional E2 sites shown (purple circles). In BPV1, the Minichromosome Maintenance Element (MME) is required for maintenance replication and genome partitioning. In HPV18, a region named the Minichromosome Maintenance Enhancer Element (MMEE) is required for maintenance replication. In HPV18, E2 binding sites #1 and #2 overlap binding sites for Sp1 and TBP (TATA) in the early promoter and thus E2 binding represses transcription. AP1 sites in the URR are important for both transcription and replication of the HPV genome. These cellular binding sites are indicated by light purple squares. Ori: origin of replication; P_E_: Early Promoter. **(B)** The structure of the HPV E2-TA protein is shown. The HPV16 transactivation domain is shown bound to the Brd4 CTM peptide (residues 1,343–1,362 in green) from the pdb file 2NNU. The E2 residues important for this interaction (R37 and I73) are shown in green. The dimeric HPV18 DNA binding domain bound to an E2 binding site is from the pdb file 1JJ4. E2 Residues that contact the Brd4 N-terminal regions (R307 and K308) are highlighted in green. An unstructured, flexible linker connects the E2 domains and is named the hinge.

The E2 proteins (E2-TA and E8^E2) are the key HPV transcriptional regulators (McBride, 2013). The E2-TA (transactivator) protein is encoded by the entire E2 open reading frame and consists of an N-terminal “transactivation” domain of about 200 amino acids joined by a flexible linker to a C-terminal DNA binding domain of ∼100 amino acids (Figure 3). The E2 protein dimerizes through the DNA binding domain and binds to specific sequence motifs (ACCN_6_GGT) in the viral genome. The E8^E2 protein also contains the flexible linker and DNA binding domain but a short 11 amino acid peptide from the E8 open reading frame is fused to the N-terminus (Dreer et al., 2017).

E2-TA can both activate and repress viral transcription, while E8^E2 functions primarily as a repressor of both transcription and replication (Dreer et al., 2017). E8^E2 binds to the E2 binding sites in the URR and represses viral transcription by recruitment of NCoR/SMRT corepressor complexes by the E8 moiety. E2-TA is a classic DNA binding transcriptional regulator that recruits cellular factors to the viral promoters (Liu et al., 2020). E2-TA can enhance transcription from heterologous promoters when E2 binding motifs are placed upstream, but the organization of the E2 binding sites in the URR is such that E2-TA can either repress or activate transcription, depending on the location of the E2 binding sites with respect to other elements. Figure 3 shows a diagram of the E2 binding sites in the URR of an oncogenic Alphapapillomavirus genome. Binding sites #1 and #2 overlap the essential transcription factor binding elements for Sp1 and TBP binding, and therefore binding of E2 to these sites inhibits transcriptional initiation (Bernard et al., 1989; Thierry and Howley, 1991). However, binding per se is not sufficient for transcriptional repression and the transactivation domain of E2 is also required (Dowhanick et al., 1995). This eventually led to the identification of Brd4 as a major E2-dependent repressor of the HPV early promoter (You et al., 2004; Wu et al., 2006).

### Different Stages of Human Papillomaviruses DNA Replication

The persistent, differentiation-dependent infectious cycle of papillomavirus relies on several phases of viral genome replication (Figure 1). The HPV E1 and E2 proteins are essential for viral DNA replication and bind to the origin of replication in the URR. E1 is a DNA helicase that specifically binds to, and unwinds, the viral replication origin to initiate DNA synthesis by cellular replicative processes. E2 functions as a helicase loader by cooperatively binding to E1 and to E2 DNA binding motifs in the origin. Both proteins recruit and associate with multiple cellular replication proteins to facilitate viral DNA replication (Bergvall et al., 2013; McBride, 2013). The E2 protein has additional roles in the maintenance phase of replication in tethering the viral genome to host chromatin (Coursey and McBride, 2019).

Upon viral entry, the virion is trafficked to the nucleus and uncoated in the vicinity of PML-nuclear bodies (Guion et al., 2019). A few rounds of viral DNA replication are required to amplify the viral genome to a low copy number. This necessitates locating in euchromatic regions of the nucleus to avoid transcriptional silencing (Bestor, 2000). The viral genomes must also become “established” in the host cell nucleus: this requires evading cellular sensors that detect foreign DNA (Della Fera et al., 2021).

Once established in the nucleus, the viral DNA replicates in S-phase concomitantly with cellular DNA replication and this results in a stable genome copy number over multiple cell generations. There has been debate about whether genomes are licensed and replicated just once per cell cycle, or whether individual genomes can be replicated several times using a random choice mechanism (Hoffmann et al., 2006). It appears that both modes can occur, with higher levels of E1 switching replication to a random choice genome amplification. There is also evidence that in some circumstances genomes can replicate in the absence of E1, and this would necessitate initiation of DNA synthesis by cellular proteins (Kim and Lambert, 2002; Egawa et al., 2012; Murakami et al., 2019). The E2 protein is important at this stage as it regulates transcription and tethers viral genomes to chromosomes as well as participating in replication initiation (Skiadopoulos and McBride, 1998; Coursey and McBride, 2019).

The last stage of viral genome replication is productive DNA amplification and occurs in differentiated cells. This serves to produce very high levels of viral DNA to be packaged into virions. Differentiated cells that amplify viral DNA are in a G2-like phase of the cell cycle and do not contain S-phase replicative machinery (Peh et al., 2002; Nakahara et al., 2005; Wang et al., 2009). Instead, they induce a DNA damage signaling response that recruits factors required for DNA synthesis and repair to nuclear replication foci (Fradet-Turcotte et al., 2009; Moody and Laimins, 2009; Fradet-Turcotte et al., 2011; Sakakibara et al., 2011; Reinson et al., 2013; Moody, 2017). The mechanism of viral DNA replication switches from a bidirectional theta mode to a recombination-directed replication mode that uses cellular homologous recombination replication processes (Gillespie et al., 2012; Sakakibara et al., 2013b).

## Interaction of the Papillomavirus E2 Protein With Bromodomain-Containing Protein 4

### Bromodomain-Containing Protein 4 Protein Structure and Function

The BET protein Brd4 functions as a scaffolding factor that binds to acetylated chromatin through its tandem bromodomains and promotes transcriptional initiation and elongation by recruiting Mediator and pTEFb complexes, respectively. In addition, Brd4 can activate transcription by directly phosphorylating RNA polymerase II (Devaiah et al., 2012), and by evicting nucleosomes and chaperoning the elongating RNA polymerase II complex through hyperacetylated histones (Kanno et al., 2014; Devaiah B. N. et al., 2016). Since its initial discovery as the Mitotic Chromatin Associated Protein, MCAP (Dey et al., 2000), the renamed Brd4 protein has been described in ∼2000 publications and has been shown to be involved in processes that reach well beyond transcription, such as DNA repair and cell reprogramming (Donati et al., 2018).

**FIGURE 4:**
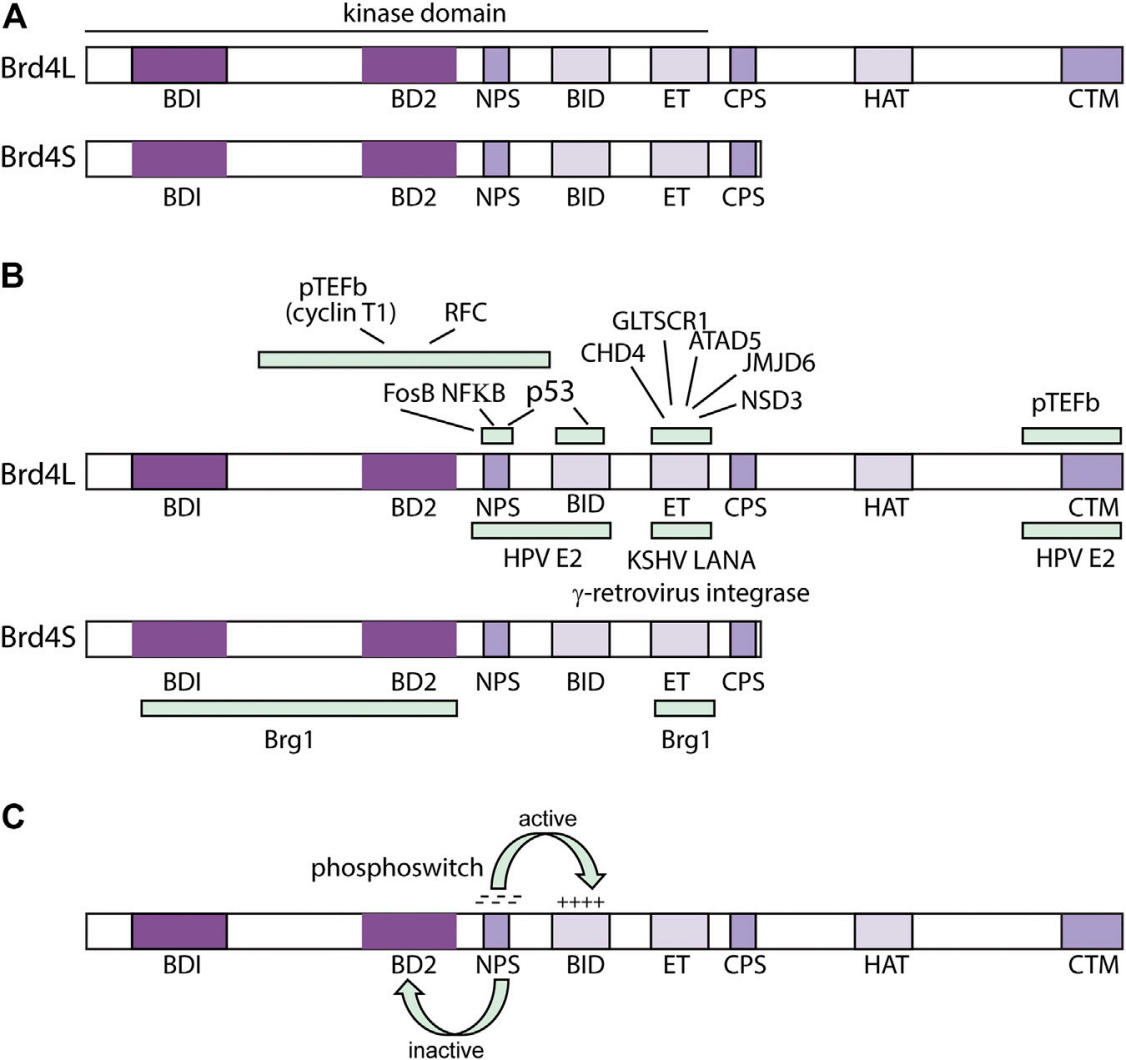
Structure and Function of the Brd4 protein. **(A)** Domains of the Brd4 protein. BRD4L and Brd4S domains are shown: BD1 (bromodomain I: residues 58–169); BD2 (bromodomain 2: residues 349–461); NPS (N-terminal cluster of phosphorylation sites: residues 484–503); BID (basic residue enriched interaction domain: residues 524–579); ET (extra-terminal domain: residues 600–678); CPS (C-terminal cluster of phosphorylation sites: residues 699–717), HAT (HAT catalytic domain: residues 1122–1161); CTM (C-terminal motif: residues 1,325–1,362) from (Devaiah B. N. et al., 2016). **(B)** Regions of Interaction of Brd4 with viral and cellular factors. From (Bisgrove et al., 2007; You et al., 2004; You et al., 2006; Wu and Chiang, 2007; Rahman et al., 2011; Sharma et al., 2013; Wu et al., 2013; Wu et al., 2016). **(C)** Brd4 Phosphoswitch. Phosphorylation of the NPS region by CK2 activates Brd4 by unmasking BD2. This also regulates interaction with the HPV E2 proteins (see text for details). From (Wu et al., 2013; Chiang, 2016; Wu et al., 2016).

There are three isoforms of the Brd4 protein (Floyd et al., 2013). They are termed Brd4L (long), Brd4S (short), and Brd4S-B (short B). Brd4L is a multi-domain protein of 1,362 amino acids in length. The two shorter forms of Brd4 are colinear with the N-terminal half of Brd4L; Brd4S is 722 residues long and only the last three residues (GPA) are unique. A minor isoform, Brd4S-B contains 75 unique C-terminal amino acids (Floyd et al., 2013). Brd4L and S are the major isoforms and the major domains of these proteins are shown in Figure 4A.

The N-terminal region of all isoforms contains two tandem bromodomains (BD1 and BD2) that bind acetylated lysines in histone tails (Dey et al., 2003). All isoforms also contain an ET (Extraterminal) domain] that activates transcription by interaction with chromatin binding factors ATAD5, CHD4, GLTSCR1, JMJD6, and NSD3 (Rahman et al., 2011). All three isoforms also contain a region known as NPS, (N-terminal phosphorylation sites) that contains seven CK2 (casein kinase II) phosphorylation acceptor sites and CPS (C-terminal phosphorylation sites) with six CK2 consensus sites (Wu et al., 2013). CPS was previously called the SEED domain due to the enrichment of S, E and D amino acids in the CK2 consensus motif (S/TxxD/E). Also, in the N-terminal half of Brd4, and present in all three isoforms, is the BID (basic interaction domain), rich in lysine residues.

Unique to Brd4L, is the CTM (C-terminal motif). The CTM constitutes the last 20 amino acids of Brd4L, which interacts with pTEFb to promote phosphorylation of RNA PolII (Jang et al., 2005). The papillomavirus E2-TA protein also interacts with the CTM (You et al., 2004; McPhillips et al., 2006; Yan et al., 2010) (Figure 4B).

Phosphorylation of Brd4 by CK2 (and dephosphorylation by PP2A) regulates intramolecular interactions among the different domains (Figure 4C). When unphosphorylated, the NPS domain can interact with bromodomain 2 (BD2) and impede binding to acetylated chromatin (Wu et al., 2013). Phosphorylation of NPS by CK2 switches its interaction from BD2 to the basic interaction domain (BID) and this allows the bromodomains to bind acetylated chromatin and Brd4 to recruit the Mediator coactivator complex (Wu et al., 2013). The NPS and BID regions can also interact intermolecularly, resulting in phosphorylation-mediated dimerization of Brd4 (McAlister et al., 2021). Less is known about the role of phosphorylation of the CPS (SEED) domain (Chiang, 2016).

### Interaction of the E2 and Bromodomain-Containing Protein 4 Proteins

The importance of the Brd4 in the papillomavirus life cycle was discovered in a quest to identify the E2 mitotic chromatin adapter involved in partitioning viral genomes, and the cellular corepressor that bound to E2-TA to repress transcription. Brd4 was copurified with and identified as a major binding factor of E2-TA (You et al., 2004), and was identified as the major factor in a protein complex that could repress E2-mediated transcription *in vitro* (Wu et al., 2006). Brd4 was also shown to bind mitotic chromosomes in complex with many viral E2 proteins (You et al., 2004; Baxter et al., 2005; McPhillips et al., 2005).

Early studies of E2 and Brd4 demonstrated that all E2 proteins tested interacted with the extreme C-terminus of the long isoform of Brd4 (Brd4L). This interaction is mediated by two highly conserved residues in the E2 transactivation domain (R37 and I73) that had previously been shown to be important for the transcription function of E2 (Senechal et al., 2007). These residues are highly conserved in all papillomaviruses sequenced to date (665 viruses, https://pave.niaid.nih.gov) with 100% E2 proteins containing R or K residues at position 37 (93%:R/7%: K) and >98% having I at residue 73. The papillomavirus hosts range from bony fish to humans and this conservation demonstrates that the Brd4-E2 interaction has been conserved for millions of years. The E2 transactivation domain forms a “cashew” shaped structure with an N-terminal bundle of three alpha helices linked to a C-terminal region of asymmetric beta-sheets by a quasihelical linker (Abbate et al., 2006). The Brd4 peptide (residues 1,343–1,362) straddles two of the alpha-helices contacting R37 and I73 residues therein (Abbate et al., 2006) (Figure 3).

Subsequent studies have identified an additional region of interaction of the E2 proteins with the N-terminal half of Brd4 (Wu et al., 2006; Wu et al., 2016). This interaction is mediated by the Brd4 BID and/or phosphorylated NPS regions, which interact with the end of the DNA recognition helix in the E2 DNA binding domain. All E2 proteins tested (BPV1 (Bovine papillomavirus), HPV16 and HPV18) interact with the accessible BID domain in the inactive form of Brd4 in which the NPS region masks BD2. However, only HPV16 and HPV18 E2 interact with the phosphorylated NPS in the active form of Brd4 (Wu et al., 2016). The significance of these differences is not yet clear with respect to the role of Brd4 in the infectious cycle of the different HPVs. These results imply that E2 proteins can interact with activated forms of both Brd4L and Brd4S. Yigitliler et al. demonstrate that HPV16 and HPV31 E2 proteins associate with Brd4S in live cells, though this interaction is mediated by the transactivation domain and hinge regions of E2, not the DNA binding domain (Yigitliler et al., 2021).

Genetic screens, and proteomic approaches have also been used to identify, and compare, Brd4 binding among papillomavirus E2 proteins from different genera of papillomaviruses. Using a genetic screen, Muller et al., showed that all 12 E2 proteins tested (from HPV1, 3, 5, 6, 8, 9, 11, 16, 18, 32, 33, and 39) interacted with the C-terminus of Brd4 (Muller et al., 2012). A proteomic study showed that 11 E2 proteins from diverse phylogenetic groups (HPV1, 8, 11,16, 18, 31, Bovine PV1, Canine PV1 and PV2, Sylvilagus floridanus PV1 and Macaca mulata PV1) also interacted with the CTM of Brd4. The latter studied showed that the interaction of Brd4 with E2 proteins from the Alpha genus (HPV11, 16, 18, 31 and MmuPV1) was much weaker than that of the other E2 proteins, and mutation of residues R37/I73 greatly reduced binding (McPhillips et al., 2006). Senechal et al. demonstrated Brd4 binding to HPV1, 11, 16, 18, 31, BPV1, and SfPV1 E2 and confirmed a reduction in binding by R37/I73 substitutions in HPV11, HPV31 and Sylvilagus floridanus PV1 (Senechal et al., 2007).

The E2 proteins have a relatively short half-life, but several studies have shown that Brd4 inhibits proteosomal degradation and increases the stability of the E2 protein (Gagnon et al., 2009; Lee and Chiang, 2009; Zheng et al., 2009; Li et al., 2014). This is likely due to the increased association of the E2-Brd4 complex with host chromatin, as described below.

Although E2 residues R37 and I73 are highly conserved, mutation of these residues gives rise to sometimes varying phenotypes that depend on the papillomavirus type, the exact amino acid substitution, and the cell type and assay that was used. The most informative mutations are those with the most conservative amino acid substitution that abrogate Brd4 binding (e.g., I73L). Table 1 lists the phenotypes of E2 mutations that affect Brd4 binding in a wide range of assays.

**TABLE 1:** Phenotypes of Papillomavirus E2 Mutations that Affect Brd4 Binding.

Domain	Virus	Mutation	Brd4-dependent transcription	Brd4 CTM binding	Brd4 pNPS binding	Transient replication	E1 binding	Extra-chromosomal replication	References
WT		WT	+++	+++	+++	+++	+++	+++	
Transactivation domain	BPV1	R37K	+++			+++,+++	++		(Brokaw et al., 1996; Zheng et al., 2005)
	BPV1	R37A	+,−,++	+		++,+,+++	+++		(Abroi et al., 1996; Abroi et al., 2004; Baxter and McBride, 2005; Baxter et al., 2005)
	CRPV	R37K	+++	++		+++			(Jeckel et al., 2002; Senechal et al., 2007)
	CRPV	R37A	+	+/−		+			(Jeckel et al., 2002; Senechal et al., 2007)
	HPV11	R37K	+	−					(Cooper et al., 1998; Senechal et al., 2007)
	HPV11	R37A	+						Cooper et al. (1998)
	HPV16	R37A	−	+/−		+,+	+++		(Sakai et al., 1996; Schweiger et al., 2006; Wang et al., 2013; Gauson et al., 2015)
	HPV31	R37K	−	+		+++		+,+++	(Stubenrauch et al., 1998; Senechal et al., 2007; Sakakibara et al., 2013)
	HPV18	R41A*		+/−		++			McKinney et al. (2016)
	BPV1	I73N	−			+++			(Ferguson and Botchan, 1996)
	BPV1	I73L	+			+++,+++	+++		Brokaw et al. (1996)
	BPV1	I73A	++,−	+		++,+++	+++		(Baxter and McBride, 2005; Baxter et al., 2005)
	CRPV	I73L	+	++		++			(Jeckel et al., 2002; Senechal et al., 2007)
	HPV16	I73A	−	−		+++, +	+++	−	(Sakai et al., 1996; Schweiger et al., 2006; Wang et al., 2013; Gauson et al., 2015)
	HPV31	I73L	−	−		+++		+++,+++	(Stubenrauch et al., 1998; Senechal et al., 2007; Sakakibara et al., 2013)
	HPV31	I73A						+++	Gauson et al. (2015)
	HPV18	I77A*		+/−		++			McKinney et al. (2016)
DNA binding domain	HPV16	K299L			+++				Wu et al. (2016)
		R302L			+/−				Wu et al. (2016)
		R304L			+++				Wu et al. (2016)
		K306N			−				Wu et al. (2016)
		K307D			+				Wu et al. (2016)
	HPV18	R307N, K308D			−				Wu et al. (2016)

## Role of Bromodomain-Containing Protein 4 in the Human Papillomaviruses Infectious Cycle

### Viral Entry

For the most part, E2 functions as a Brd4-dependent transcriptional repressor of the viral early promoter in the context of the HPV genome. However, Brd4 activates both early viral transcription and replication when the HPV18 genome is delivered in a virion particle and mutations in the E2 CTM contact residues R37/I73 have little effect (McKinney et al., 2016). Thus, Brd4 is an activator of viral transcription at very early stages of infection before the E2 protein is expressed and converts Brd4 to a transcriptional repressor (McKinney et al., 2016). Concordantly, BET bromodomain inhibitors reduced viral transcription upon HPV11 infection, also demonstrating that Brd4 is an activator at this stage of infection (Morse et al., 2018). Notably, the histones that are assembled on viral genomes and packaged in viral capsids are enriched in acetylated residues that could potentially function to enhance early transcription and recognition by Brd4 (Porter et al., 2021).

### Transcriptional Regulation

The viral E2 proteins activate transcription of heterologous promoters, as well as those of animal papillomaviruses such as BPV1, in a Brd4-dependent manner (McPhillips et al., 2006; Schweiger et al., 2006). This is, at least in part, due to recruitment of the Mediator complex and pTEFb to the promoter. Brd4 also recruits NSD3, JMJD6, and GLTSCR1 through interactions with the ET domain and these contribute to transcriptional activation in a pTEFb independent manner (Rahman et al., 2011). Brd4 and E2 also activate viral transcription indirectly (Delcuratolo et al., 2016); Brd4 and the SfPV1 E2 protein can upregulate c-fos, which in turn promotes viral transcription through AP1 sites in the URR (Delcuratolo et al., 2016) (Figure 3). Brd4 can also promote viral transcription by tethering the E2 protein to transcriptionally active regions of chromatin (Jang et al., 2009; Helfer et al., 2014).

In the Alphapapillomavirus HPVs, the E2-TA protein activates viral transcription by binding to sites distal to the promoter (E2 binding site #4), but for the most part they repress transcription by binding to proximal sites that overlap essential promoter elements (E2 binding sites #1 and #2) (Figure 3). These E2 sites overlap binding sites for Sp1 and TBP and binding of E2 blocks recruitment of these factors (Thierry and Howley, 1991; Demeret et al., 1994; Demeret et al., 1997). However, E2-mediated transcriptional repression also requires the transactivation domain, and in particular the Brd4 contact residues R37/I73, implicating Brd4 in E2-mediated transcriptional repression (Wu et al., 2006; Schweiger et al., 2007; Smith et al., 2010). E2-Brd4 mediated repression of the HPV early promoter is dependent on the histone acetyl transferase Tip60 (Jha et al., 2010). Tip60 facilitates transcriptional repression by acetylating multiple lysine residues in histone H3 K14 and K5, K8, K12, and K16 in histone H4 in the early viral promoter, which are bound by the Brd4 bromodomains (Jha et al., 2010; Smith et al., 2010; Filippakopoulos and Knapp, 2012).

Brd4L recruits pTEFb to promoters to promote phosphorylation of RNA polymerase II and transcriptional elongation (Jang et al., 2005; Itzen et al., 2014). This interaction is mediated by the C-terminal region of Brd4L, which also interacts with the E2 protein (Bisgrove et al., 2007). The E2 protein can disrupt the interaction of Brd4L and pTEFb, leading to transcriptional repression (Yan et al., 2010), and a fragment of the Brd4 C-terminal region acts as a dominant negative and inhibits E2-mediated repression (Yan et al., 2010). However, other studies find that the Brd4 CTD interferes only with E2-mediated transactivation and not repression (Smith et al., 2010). Notably, the TAT protein of HIV also interacts with pTEFb to activate viral transcription and this is inhibited by the C-terminal region of Brd4L (Bisgrove et al., 2007). Therefore, in both HPV and HIV infection Brd4 activates basal transcription of the viral early promoter, but competes with the viral trans activator for pTEFb binding and thus represses activated transcription (Ott et al., 2011).

Brd4S represses HIV transcription by recruiting the Brg1 component of the SWI/SNF complex to the HIV promoter resulting in repressive chromatin architecture (Conrad et al., 2017). Brd4S interacts with HPV16 and HPV31 E2 proteins in cells with extrachromosomal viral genomes and represses late viral transcripts in undifferentiated cells (Yigitliler et al., 2021). Notably, the cervical carcinoma derived C33-A cells used in many HPV studies are deficient in Brg1 and this could explain conflicting results obtained in different cell types (Kutluay et al., 2009).

Brd4 is clearly a key player in the regulation of HPV transcription, but many details must still be elucidated. Viral transcription can be modulated by E2-TA levels (determines which E2 sites are bound), E8^E2 levels, the balance of Brd4L and Brd4S isoforms, and post-translational modifications of viral and cellular factors. Indirect activation of cellular transcription factors adds another layer of complexity. Overlaying transcriptional regulation is replication of the extrachromosomal HPV genome as the origin of replication overlaps the early promoter elements (Figure 3). The complexity of this regulation allows the virus to respond to the varying cellular environment to promote the persistent HPV infectious cycle.

### Viral DNA Replication: Initiation of DNA Synthesis

HPV replication is initiated by binding of the viral E1 and E2 proteins to the replication origin, allowing cellular enzymes to duplicate viral DNA in a bidirectional theta mode. However, the precise role of Brd4 in HPV replication is not clear. In many studies, E2 proteins mutated in either R37 or I73 cannot bind Brd4 but can support transient DNA replication (Brokaw et al., 1996; Sakai et al., 1996; Cooper et al., 1998; Baxter et al., 2005; Ilves et al., 2006; Schweiger et al., 2006; Senechal et al., 2007; Gauson et al., 2015). But others find that E2 proteins mutated in both R37/I73 are partially defective in replication (Wang et al., 2013). Downregulation of Brd4 expression can reduce HPV replication but since this affects cell cycle, it is difficult to conclude that this is direct (Wang et al., 2013).

Brd4 has consistently been shown to be associated with the replication foci that form when cells containing HPV genomes are differentiated (Sakakibara et al., 2013a), or when E1 and E2 proteins are transiently expressed at high levels in undifferentiated keratinocytes (Sakakibara et al., 2013a; Wang et al., 2013; Gauson et al., 2015; Yigitliler et al., 2021). In the latter case, the foci recruit and are dependent on Brd4 even in the absence of a viral replicon (Sakakibara et al., 2013a). Although these E1-E2 foci form in undifferentiated cells, they likely represent the late foci that develop in differentiated cells in response to high levels of E1 and E2 protein and they will be discussed more in the next section.

The formation of nuclear foci promotes viral replication by recruiting and concentrating essential cellular components of the replication machinery at late stages of infection. However, this Brd4-enrichment of factors could also be important for replication at early stages of infection, when the E1 and E2 proteins are expressed in very limited quantities. In support of this hypothesis, Gauson and colleagues show that while a Brd4 binding defective E2 protein (R37A) supports replication at levels close to wildtype when expressed at high levels, it becomes very defective at low levels (Gauson et al., 2015).

### Viral DNA Replication: Maintenance and Partitioning of Viral Genomes

The E1 and E2 proteins support transient DNA replication by initiating DNA synthesis at the viral replication origin, but this is not sufficient for the long-term maintenance replication that is characteristic of papillomavirus genomes (Piirsoo et al., 1996). Studies in BPV1 demonstrated that regions of the viral URR containing additional E2 binding sites (named the Minichromosome Maintenance Element; MME) are also required in addition to the replication origin (Piirsoo et al., 1996) (Figure 3). The observation that both the E2 protein and viral genomes associated with mitotic chromosomes led to the model in which the E2 protein tethers viral genomes to host chromosomes to retain and partition the viral genomes in dividing cells (Skiadopoulos and McBride, 1998). The transactivation domain of E2 associates with host chromatin while the DNA binding domain binds to the E2BS in the viral genome (Bastien and McBride, 2000; Abroi et al., 2004).

Subsequent studies identified Brd4 as an important target of the E2 protein-viral genome complex on mitotic chromosomes (You et al., 2004; Baxter et al., 2005; McPhillips et al., 2005; Ilves et al., 2006). E2 greatly stabilizes the association of Brd4 with chromatin, and Brd4 and E2 colocalize in small punctate foci on mitotic chromosomes (McPhillips et al., 2005; Li et al., 2014). Mutation of R37/I73 residues abrogate binding of E2 and Brd4 on mitotic chromosomes (Baxter et al., 2005; McPhillips et al., 2005; You et al., 2005; Ilves et al., 2006) and expression of the dominant negative Brd4 CTD disrupted E2/Brd4 binding and resulted in loss of BPV1 genomes from cells (You et al., 2005).

The E2-Brd4 tethering model is well established for BPV1; BPV1 E2 and Brd4 interact with high affinity and there are multiple E2 binding sites in the URR of the viral genome (Figure 3). The situation is not so clear for other papillomaviruses, particularly the human Alphapapillomaviruses. As described above, all papillomavirus E2 proteins tested bind Brd4 but with different affinity and not all are detected as readily on mitotic chromosomes as BPV1 E2 (McPhillips et al., 2006; Oliveira et al., 2006; Jang et al., 2015). Brd4 and E2 proteins from Delta (BPV1), Mu (HPV1) and Kappa (OcPV1 and SfPV1) genera interact strongly, and colocalize on interphase and mitotic chromatin (McPhillips et al., 2006; Oliveira et al., 2006). The Betapapillomavirus E2 proteins bind strongly to Brd4 but localize to the pericentromeric regions of mitotic chromosomes (Oliveira et al., 2006; Poddar et al., 2009). This interaction is mediated by a short peptide in the hinge region of E2 that is phosphorylated by PKA (protein kinase A). When this motif is mutated, the Betapapillomavirus E2 proteins revert to binding on the arms of mitotic chromosomes with Brd4 like other E2 proteins (Sekhar et al., 2010; Sekhar and McBride, 2012; McBride, 2013).

The role of Brd4 in tethering Alphapapillomavirus genomes to chromosomes is still somewhat elusive. The Alphapapillomavirus E2 proteins bind to Brd4 *in vitro* with lower affinity than other E2 proteins, though this could potentially be modulated *in vivo* by the additional phosphorylation dependent interactions between the E2 proteins and the BID and NPS regions of Brd4 described above. When stably expressed in cells, the Alphapapillomavirus E2 proteins are not readily detected on mitotic chromosomes like other papillomavirus E2 proteins except in late telophase (Oliveira et al., 2006; Donaldson et al., 2007). This could be due to the sensitivity of detection as HPV16 E2 can be detected on chromatin by bimolecular fluorescence complementation, by overexpression of a GFP-E2 fusion protein, or by ChIP when E1 and E2 are coexpressed (Helfer et al., 2013; Chang et al., 2014; Jang et al., 2014). Furthermore, Brd4 is strongly recruited to nuclear foci that result from Alphapapillomavirus HPV E1 and E2 expression (Sakakibara et al., 2013a; Wang et al., 2013).

To identify the regions of mitotic host chromatin bound by E2-Brd4 complexes, Jang and colleagues performed chromatin immunoprecipitation in combination with microarray (ChIP on chip) in C33-A cells expressing HPV1 E2 (Jang et al., 2014). HPV1 is a Mupapillomavirus and was used in this study because of the high affinity interaction of HPV1 E2 and Brd4, and the distinct punctate speckles of the E2-Brd4 complex on mitotic chromosomes (Oliveira et al., 2006). This revealed that the E2-Brd4 complex bound to large domains of active chromatin, rich in histone acetylation and histone H3 K4me1, some of which were related to common fragile sites (Jang et al., 2014). Brd4 was already present at some of these sites (though undetectable by immunofluorescence), but chromatin binding was greatly enhanced and stabilized in the presence of HPV1 E2. HPV16 E1 and E2 were also shown to bind to similar sites in C33-A cells when expressed transiently. Moreover, HPV replication foci frequently formed adjacent to these regions, leading to the proposal that Brd4 nucleated HPV replication foci at sites susceptible to replication stress (Jang et al., 2014).

One consideration in our understanding of the role of Brd4 in the HPV lifecycle, is that many experiments in the HPV field are carried out in C33-A cells. C33-A cells are unusual and invaluable in that they will tolerate continual low level expression of viral E2 proteins (McPhillips et al., 2006). However, as described above, they are deficient in the Brd4 associated protein, Brg1 (Kutluay et al., 2009) and this could contribute to the accumulation of Brd4 in the observed chromatin speckles at regions of replication stress (Jang et al., 2014). The studies of Brd4 in C-33A cells stably expressing the E2 proteins provides important insight into the role of Brd4 in the HPV lifecycle, however some contradictory results could be explained by the use of different cells.

It is very difficult to analyze E2 mutations that abrogate binding in the background of the HPV genome because E2 is involved in so many processes and most mutations result in defective genomes (Gauson et al., 2015). However, HPV31 E2 proteins with conservative substitutions R37K or I73L can replicate extrachromosomally even though they are unable to activate transcription or bind to Brd4 *in vitro* or *in vivo* (Stubenrauch et al., 1998; Senechal et al., 2007; Sakakibara et al., 2013a). Is this sufficient evidence to conclude that Brd4 is not required for replication or partitioning of HPV31 genomes? Perhaps other E2-Brd4 interactions compensate for the disruption of the interaction of the Brd4 CTM with the E2 transactivation domain, or other viral or cellular proteins complement the defective E2-Brd4 interaction.

Another notable difference in Alphapapillomavirus genomes is the paucity of E2 binding sites in the URR (McBride, 2013). Most Alphapapillomavirus genomes contain just four E2 binding sites: three in the origin/early promoter region and one further upstream in the URR (Figure 3). This 4^th^ distal site is not required for long-term replication of URR containing replicons and instead a region containing the transcriptional enhancer is required (Van Doorslaer et al., 2017; Coursey et al., 2021). Furthermore, only two E2 binding sites are required to segregate HPV18 derived plasmids in the absence of replication (Ustav et al., 2015). These studies support the involvement of E2 in maintenance replication and partitioning, but likely additional factors are required to stabilize the tethering complex. Taken together, these studies indicate that the E2 and Brd4 interaction is important for maintenance of Alphapapillomavirus genomes but might be more complex than for other papillomaviruses.

### Viral Genome Amplication and Late Gene Transcription

As described above, nuclear replication foci form in differentiated keratinocytes harboring extrachromosomal HPV genomes, or in keratinocytes transfected with E1 and E2 expression vectors (Fradet-Turcotte et al., 2011; Sakakibara et al., 2011; Reinson et al., 2013). Many factors associated with the DNA damage response are recruited to these foci and this allows HPV to replicate to high levels in differentiated cells that are no longer in S-phase, most likely by a recombination-directed replication mechanism (Sakakibara et al., 2011; Gillespie et al., 2012; Sakakibara et al., 2013b). As yet, there is no direct evidence that Brd4 participates directly in the late viral DNA replication process but there is increasing evidence that Brd4 can participate in DNA replication and repair (Zhang et al., 2018; Kang et al., 2019; Kim et al., 2019; Wessel et al., 2019; Lam et al., 2020; Song et al., 2020).

Sakakibara et al. showed that nuclear foci that form in keratinocytes transfected with Alphapapillomavirus E1 and E2 expression vectors depend on the ability of E2 to bind Brd4 and are disrupted by inhibitors that displace Brd4 from chromatin (Sakakibara et al., 2013a). Moreover, in the presence of an origin containing replicon Brd4 is no longer required for focus formation and is not recruited into foci (Sakakibara et al., 2013a). On the contrary, Wang et al. find that in C33-A cells Brd4 is only recruited to replication foci in the presence of a replicating HPV genome (Wang et al., 2013). Gauson et al. also observe Brd4 in foci replicating HPV genomes and show that they can be disrupted by shRNA downregulation of Brd4 expression (Gauson et al., 2015). These differences could be due to the presence of small pre-existing Brd4 foci in C33-A cells that represent binding to large regions of chromatin, some of which are related to common fragile sites (Jang et al., 2014). Of note, both Brd4S and Brd4L are recruited to these foci in C33-A cells (Yigitliler et al., 2021).

Brd4 forms a satellite pattern around replication foci that are amplifying viral DNA in differentiated keratinocytes (Sakakibara et al., 2013a). Based on the findings described above, it was proposed that the association of viral genomes with Brd4 was an early event in the HPV infectious cycle that served to associate viral genomes with beneficial host chromatin and partition them to dividing cells; upon genome amplification Brd4 would be displaced to the periphery of the foci as the replication foci expanded (Sakakibara et al., 2013a). Another hypothesis, based on the finding that the Brd4 isoform, Brd4S(B) insulated chromatin from the DNA damage response (Floyd et al., 2013), was that Brd4 insulated host DNA from the strong DNA damage signaling occurring in the replication foci (Sakakibara et al., 2013a). However, Brd4S(B) is not expressed well in keratinocytes (unpublished data). A third possibility is that Brd4 is regulating viral transcription on the surface of the replication foci (Khurana and McBride, 2021) and there is some evidence that Brd4 might activate the late promoter (Songock et al., 2017). Lastly, Brd4 could prevent the accumulation of R-loops that occur due to clashes in transcription and replication (Edwards et al., 2020; Lam et al., 2020).

## Integration of Viral Genomes in Human Papillomaviruses-Associated Cancers

As described throughout this article, papillomaviruses replicate as extrachromosomal genomes in the nucleus of infected cells. However, sometimes the viral genomes become accidently integrated into the host chromosomes (McBride and Warburton, 2017). In most cases this would be inconsequential, but occasionally an oncogenic HPV integrates in such a way that expression of the E6 and E7 oncogenes becomes dysregulated and this can drive carcinogenesis. In fact, HPV genomes are integrated in the majority of HPV-associated cancers (Pett and Coleman, 2007). Integration of the viral genome often disrupts the E2 gene and relieves repression of the early viral promoter. Thus, this integration event can change the role of Brd4 from transcriptional repressor to transcriptional activator.

In some cases, the copy number of the integrated viral genome can be amplified *in situ* resulting in tandem arrays of the viral genome, with or without intervening cellular sequences (McBride and Warburton, 2017). In one example of cells derived from a cervical lesion, the HPV16 integration event captured an adjacent cellular enhancer and amplified both viral and cellular sequences over 20 times to create a Brd4-dependent super-enhancer (Dooley et al., 2016; Warburton et al., 2018). Treatment of these cells with BET inhibitors disrupted Brd4 binding and resulted in rapid cell senescence (Dooley et al., 2016).

Together with Brd4, the viral E2 proteins interact with host chromatin at many stages of the viral life cycle. Not surprisingly, this association can influence the sites of accidental integration. For example, the E2-Brd4 complex associates with regions of host chromatin that are susceptible to replication stress, and replication foci frequently form at these sites (Jang et al., 2014). In fact, it has been recognized for almost thirty years that HPV genomes often integrate adjacent to common fragile sites (Smith et al., 1992). Brd4 is a major component of cellular super-enhancers and transcriptional regulatory hubs. HPV integration sites are also associated with transcriptionally active regions of the host genome. Of note, some hotspots of recurrent HPV integration in the human genome are closely associated with Brd4-associated super-enhancers in HPV positive cervical keratinocytes (Warburton et al., 2021). Extrachromosomal HPV genomes may preferentially associate with such transcriptional epicenters to ensure persistent infection. However, accidental integration and clonal selection of these sites can result in oncogenesis.

## Potential of Bromodomain and Extra-Terminal Domain Inhibitors as Therapeutics for Human Papillomaviruses Infection and Associated Cancers

Many inhibitors have been developed that interfere with Brd4 function, such as bromodomain recognition of acetylated lysines (Filippakopoulos et al., 2010) or phosphorylation of activating Brd4 domains (Chiang, 2016), and these could be used in anti-viral therapies. These inhibitors are highly effective and could reduce viral transcription or disrupt tethering to cause the loss of extrachromosomal genomes. In HPV-associated cancers, Brd4 inhibition could disrupt oncogene expression leading to cell senescence, or promote sensitivity to other therapeutics (Rataj et al., 2018) and BET inhibition prevented formation of Sylvilagus floridanus PV1 induced warts(Morse et al., 2018). However, the relationship between Brd4 and HPV is multi-tiered and complicated, and care must be taken to ensure that these strategies do not backfire by promoting viral oncogene transcription or genome integration. Carefully designed combination therapies might help ensure that the virus is eliminated from infected cells.

## Conclusion

The Brd4 proteins are integral to many steps and processes in the HPV infectious cycle. These interactions are multi-tiered, and transcription, replication and genome partitioning are most likely interdependent processes. Understanding how HPVs make use of Brd4 functions to support the differentiation-dependent viral lifecycle may shed light on the cellular functions of this key regulator. In turn, the intense research on the cellular functions of Brd4 could lead to the development of anti-viral therapies.
